# The Impact of the Nitric Oxide (NO)/Soluble Guanylyl Cyclase (sGC) Signaling Cascade on Kidney Health and Disease: A Preclinical Perspective

**DOI:** 10.3390/ijms19061712

**Published:** 2018-06-09

**Authors:** Shalini M. Krishnan, Jan R. Kraehling, Frank Eitner, Agnès Bénardeau, Peter Sandner

**Affiliations:** 1 Bayer AG, Cardiovascular Research, Pharma Research Center, 42096 Wuppertal, Germany; shalini.murali@bayer.com (S.M.K.); jan.kraehling@bayer.com (J.R.K.); frank.eitner@bayer.com (F.E.); agnes.benardeau@bayer.com (A.B.); 2 Division of Nephrology and Clinical Immunology, RWTH Aachen University, 52062 Aachen, Germany; 3 Department of Pharmacology, Hannover Medical School, 30625 Hannover, Germany

**Keywords:** nitric oxide, cGMP, chronic kidney disease, soluble guanylyl cyclase, sGC, sGC stimulator, sGC activator

## Abstract

Chronic Kidney Disease (CKD) is a highly prevalent disease with a substantial medical need for new and more efficacious treatments. The Nitric Oxide (NO), soluble guanylyl cyclase (sGC), cyclic guanosine monophosphate (cGMP) signaling cascade regulates various kidney functions. cGMP directly influences renal blood flow, renin secretion, glomerular function, and tubular exchange processes. Downregulation of NO/sGC/cGMP signaling results in severe kidney pathologies such as CKD. Therefore, treatment strategies aiming to maintain or increase cGMP might have beneficial effects for the treatment of progressive kidney diseases. Within this article, we review the NO/sGC/cGMP signaling cascade and its major pharmacological intervention sites. We specifically focus on the currently known effects of cGMP on kidney function parameters. Finally, we summarize the preclinical evidence for kidney protective effects of NO-donors, PDE inhibitors, sGC stimulators, and sGC activators.

## 1. Summary

The number of patients with chronic kidney diseases has dramatically increased in recent years, driven by the constant increase in life expectancy and the steadily increasing prevalence of risk factors such as diabetes, obesity or hypertension. Besides the progressively declining kidney function harboring a poor prognosis for patients, enormous economic costs—especially for hemodialysis and kidney transplantation—cause a huge and constantly growing burden for healthcare systems. Therefore, intense research and clinical development efforts are ongoing to identify signaling pathways which are critical for maintenance of kidney function and to investigate new pharmacological interventions for the treatment of kidney diseases. In recent years, it has become obvious that, along with the natriuretic peptide (NP)/particulate guanylyl cyclase (pGC)/cyclic guanosine monophosphate (cGMP) signaling cascade, the nitric oxide (NO)/soluble guanylyl cyclase (sGC)/cGMP pathway plays a pivotal role for the regulation of physiological kidney function. The disruption of the NO/sGC/cGMP pathways results in the decrease of cGMP levels and can cause severe kidney pathologies. Within this review article, we summarize the current understanding how NO/sGC/cGMP systems regulate physiological kidney function by targeting renal blood vessels and vascular smooth muscle cells, but also by having an impact on renal innervation, glomeruli, mesangial cells, podocytes, macula densa and tubular cells. In addition, we discuss how impairment of this pathway in different functional kidney compartments leads to chronic kidney pathologies. Finally, we summarize the current pharmacological approaches, aiming to restore NO/cGMP signaling for the treatment of kidney diseases. Here, we focus on cGMP increase via NO donors, inhibition of cGMP degradation by PDE-inhibitors and direct stimulation of the soluble guanylyl cyclase (sGC) by sGC stimulators and sGC activators.

## 2. The NO/sGC/cGMP Signaling

**Figure 1:**
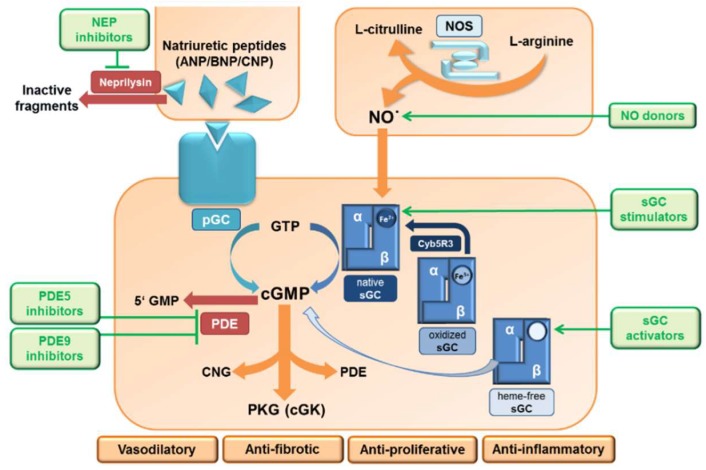
The NO/sGC and NP/pGC signaling cascade with major pharmacological intervention sites. NO/sGC- and NP/pGC-derived cGMP predominantly targets cGMP-dependent protein kinases (PKG/cGK), but also has mediates its actions though cyclic nucleotide-gated (CNG) ion channels and the activation or inhibition of phosphodiesterases (PDEs). The differential expression of enzyme isoforms in various cells and tissues, which mediate cellular effects, also have a direct impact on kidney function. The signaling cascade is currently targeted on the level of cGMP production (nitrates, sGC stimulators, and sGC activators) and cGMP degradation (PDE5 and PDE9 inhibitors). Abbreviations: ANP: Atrial natriuretic peptide; BNP: Brain natriuretic peptide; cGK: cGMP-dependent protein kinases; cGMP: cyclic guanosine monophosphate; CNG: Cyclic nucleotide-gated ion channels; CNP: C-type natriuretic peptide; GTP: cyclic guanosine triphosphate; GMP: guanosine monophosphate; NO: Nitric oxide; NOS: Nitric oxide synthase; NP: Natriuretic peptide; PDE: Phosphodiesterase; pGC: particulate guanylyl cyclase; PKG: Protein kinase G; sGC: Soluble guanylyl cyclase.

In 1998, Drs. Robert Furchgott, Louis Ignarro, and Ferid Murad were awarded the Nobel Prize in Physiology and Medicine “for their discoveries concerning NO as a signaling molecule in the cardiovascular system”. These researchers unveiled the molecular nature of the “Endothelial Derived Relaxing Factor (EDRF)” to be the gas NO, which is produced by the endothelium and potently dilates blood vessels. Further research demonstrated that NO is of broad importance not only for the regulation of vascular tone via smooth muscle cell relaxation, but this signaling system is also active in a variety of other cells types. Depending on the site of action, it influences nerve function and synaptic transmission, has antifibrotic and antiproliferative effects, influences blood clotting and inflammatory processes and is present in adipocytes with impact on fat metabolism. The signaling cascade—especially for the vasodilatory effects—is very well understood and schematically summarized in Figure 1.

**Figure 2:**
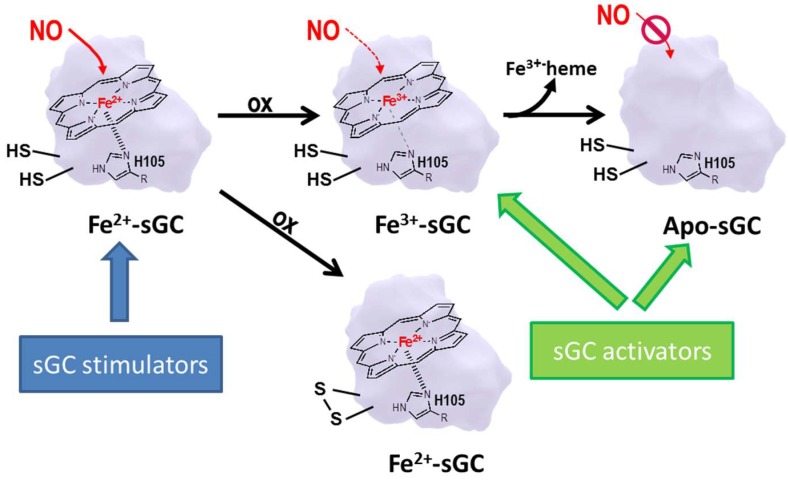
The cofactor heme is bound to sGC by various amino acids, but the central iron of heme builds a coordinative bound with the proximal histidine-105 (H105) of the sGC. Under oxidative conditions the heme iron changes to its Fe^3+^ state, resulting in a weakened sGC/heme bound and subsequently leading to the Apo form of sGC, which cannot be regulated by NO. Recent literature [1] indicates that oxidative stress can also lead to the formation of disulfide bridges normally absent in sGC and thereby affecting the function of the enzyme.

The signaling starts with the formation of NO as a by-product during the conversion of l-arginine to l-citrulline, a reaction catalyzed in the presence of the nitric oxide synthase (NOS) enzyme. There are three different NOS isoforms identified in mammals: neuronal (nNOS/NOS1), inducible (iNOS/NOS2), and endothelial (eNOS/NOS3). eNOS-derived NO stimulates soluble guanylyl cyclase (sGC)—an intracellular receptor present in effector cells—which results in the generation of cGMP from guanosine triphosphate (GTP). sGC forms a heterodimer consisting of two subunits, α and β, and NO activates sGC by binding to the heme-group present on the β-subunit. However, the heme in sGC is prone to be oxidized (from Fe^2+^ to Fe^3+^), e.g. during oxidative stress, which leads to a less tight binding between the sGC and its cofactor, subsequently leading to a heme-free form of sGC (Apo-sGC) (Figure 2), and Apo-sGC (or heme-free sGC) can no longer respond to NO and produce cGMP. In addition, NO-dependent formation of reactive oxygen species like superoxide anion causes oxidative stress and results in thiol oxidation and nitrosylation of cysteines in the sGC. This decreases its catalytic activity and thereby decreases cGMP production [1].

Recently, it has been identified that the enzyme cytochrome b5 reductase 3 (Cyb5R3) is able to reduce Fe^3+^-sGC to the native Fe^2+^-sGC form, which is then again able to respond to NO and produce cGMP [2]. The physiological effects of cGMP are predominantly mediated through the activation of cGMP-dependent protein kinases (PKG/cGK), cyclic nucleotide-gated (CNG) ion channels and the activation or inhibition of phosphodiesterases (PDEs).

The differential expression of enzymes isoforms in various cells and tissue types helps to directly regulate the relevant physiological function. In the case of NOS enzymes, as the names suggest, within the kidney, eNOS is expressed predominantly in the renal vascular endothelium but also in the tubuli, nNOS is expressed in the macula densa and in specialized neurons surrounding renal arteries and interstitial cells and although iNOS is quite lowly expressed in the normal kidney, expression increases in response to stimuli such as inflammation [3]. Sullivan et al. showed that the renal medulla expresses all three isoforms of NOS, and this compartment is known to express the highest amounts of NOS protein and activity within the kidney, allowing for regulation of medullary blood flow and sodium and water balance [4,5]. The subunits of sGC appear to be differentially expressed in different regions of the kidney, as Bachmann et al. revealed that the α_1_ subunit is expressed in glomerular podocytes, whereas the β_2_ is found in principal cells of the collecting duct [6]. The use of antibodies specific to the β_1_ subunit revealed that sGC is expressed largely in the renal vasculature and granular cells thus playing an important role in renal perfusion and renin release, respectively [7]. While both PKG-Iα and PKG-Iβ isoforms are found in the kidneys as well as the vascular smooth muscle cells, PKG-Iα is 10-fold more sensitive to cGMP than PKG-Iβ. PKG-II is also expressed in the kidney and has been shown to regulate renin release [8]. However, it is important to note that enzyme activity is regulated not only by its expression as the protein is also susceptible to post-translational mechanisms. Therefore, additional data would be necessary to link the expression profiles with respective enzyme-activity within these functional compartments.

## 3. The NPs/pGCs/cGMP Signaling

Besides the NO/sGC/cGMP signaling, there is a second major source for cGMP production within the kidney. Renal cGMP levels can also be increased via stimulation of the particulate guanylyl cyclase (pGC) by natriuretic peptides. Atrial natriuretic peptide (ANP), B-type natriuretic peptide (BNP), and C-type natriuretic peptide (CNP) are produced and secreted predominantly from cardiomyocytes and endothelial cells, especially under disease conditions such as pressure overload and heart failure, and are involved in regulating sodium and water excretion and maintaining blood pressure. NPs work in an endocrine manner and bind to membrane-bound pGC which consists of an extracellular ligand-binding domain and cytoplasmic guanylyl cyclase domain. While there are at least seven pGCs identified, some of which are known to be stimulated by multiple ligands, it is well known that ANP and BNP specifically bind to pGC A (GC-A), whereas CNP binds to pGC B (GC-B). Both GC-A and GC-B are expressed in the kidney and have been shown to be of clinical relevance. Similar to cGMP derived from stimulation of sGC by NO, the physiological effects of cGMP derived from pGC stimulation are also mediated through the three target proteins, namely PKG, CNG ion channels and PDEs. Depending on the organ system(s) involved, the binding of ANP/BNP to GC-A induces vasodilation, natriuresis, and diuresis and in addition, it also has antifibrotic, antihypertrophic, and anti-inflammatory effects [9], whereas the effects of CNP binding to GC-B largely involve antifibrotic and antiproliferative effects in the heart, vasculature and kidneys. More specifically, in the kidneys, the effects of NPs are known to increase GFR by causing vasodilation of the afferent arteriole, vasoconstriction of the efferent arteriole, and promoting sodium and water excretion by inhibition of Na^+^ channels in the tubules and collecting duct and antagonism of vasopressin in the collecting duct [10]. However, the pGC/cGMP system could not be reviewed extensively in the context of this overview, and pharmacological approaches enhancing pGC signaling are still scarce. Therefore, within this article, the focus has been put on the NO/sGC/cGMP signaling pathway. 

## 4. Physiological Function of Kidney Compartments and Regulation via NO/sGC/cGMP Signaling

**Figure 3:**
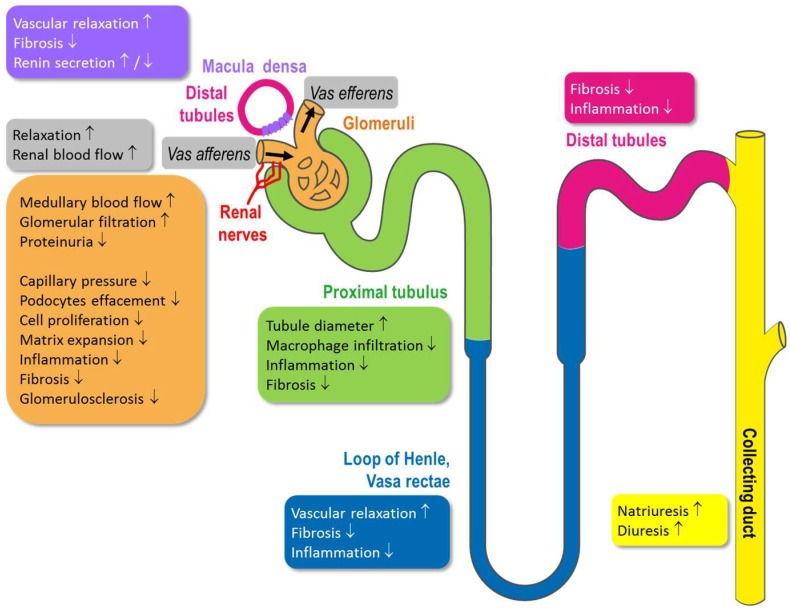
Schematic representation of a nephron and its functional units (glomerulus proximal tubulus, loop of Henle, distal tubulus and collecting duct and effects of NO/cGMP (increase/decrease: ↑/↓). (Nephron structure based on Servier Medical Art)

The kidney consists of specialized morphological structures with specific functions (Figure 3). These functional units comprise glomeruli that colocalize with afferent and efferent arterioles, which form the Bowman’s capsule. This compartment is key for the filtration of blood, and the glomeruli membrane is surrounded externally by podocytes that constitute a protective layer in addition to the glomerular endothelial cells and smooth muscles cells located on the inner surface of the vessels. Mesangial cells regulate capillary flow which directly influences the glomerular filtration rate (GFR). Afferent and efferent arterioles constrict and dilate under various stimuli and regulate glomerular blood flow. The diameter of the efferent arterioles is smaller than the afferent arterioles, thus creating background resistance to blood flow. Hydraulic pressure of the renal circulation drops progressively along intra-renal micro circulation. Pressure measured by micro punctures in afferent arterioles (of monkey and rats) was found to be superior to that of efferent arterioles [11]. The diameter and pressure of afferent arterioles is controlled by renal nerves, while NO is capable of dilating both afferent and efferent arterioles [12].

After passing the glomeruli, the tubules are important for ion and fluid retention and excretion and are also tightly regulated compartments. The proximal tubule is the structure next to glomeruli. Proximal tubules constitute more than one half of renal mass and reabsorb almost all macromolecules filtered by glomeruli under a process that requests high energy [13]. Damage to the function of proximal tubules significantly contributes to kidney disease progression [14]. Distal tubules (and macula densa) are involved in electrolytes (Na^+^, Cl^−^, K^+^ and Ca^2+^), passive or active water reabsorption, and also contribute to pH regulation. Each segment works in concert to maintain homeostasis. Tubules are peculiarly sensitive the effect of diuretics. The macula densa is a specific part of the distal tubule that is located close to the glomeruli arterioles. The macula densa not only regulates flow rate through the nephron but also sodium chloride concentration and osmolality of the tubular fluid. Its function depends ultimately on ATP. Like other organs, kidney function is also controlled by nerves that are widely connected to specific kidney structures (Figure 3). Liu et al. [15] nicely described the presence of NOS-immunoreactive neurons mainly located in the connective tissue between the renal pelvis and the renal parenchyma in the advanced stages of development. Those NOS-containing nerve fibers with vasomotor and sensitive potential are able to modulate renal functions. Additionally, the dense microcirculation formed by afferent and efferent arterioles located near the glomeruli is directly regulated by the nerves activity that regulates arterioles pressure and diameter.

## 5. Impact of the Impaired NO/sGC/cGMP Signaling on Kidney Function

The impact of sGC modulation or NO/cGMP production was first investigated in the field of cardiovascular and cardiopulmonary diseases. However, cGMP plays also a pivotal role in regulation of kidney function by direct influence on different functional kidney compartments. Therefore, growing evidence suggests a role of the NO/sGC/cGMP signaling in kidney pathophysiology and impaired cardiorenal cross-talk.

Each type of kidney disease differs from each other in many respects, such as disease etiology, damages of specific kidney structures, presence of hypertension, extent of albuminuria, time-course progression, etc. Regardless of the underlying etiology for kidney pathologies, impairment of NO/cGMP signaling could be found in the majority of them and might be an overarching strategy for treatment of a broad range of kidney diseases. As mentioned previously, cGMP could exert a broad variety of biological actions ranging from vasodilation—which is very well understood—over the less understood antifibrotic, antiproliferative and anti-inflammatory mode of actions, to effects on neurotransmission and lipolysis [16,17]. These effects could play a role in regulation of physiological kidney function but might be especially important when kidney function is impaired.

It is well established that NO/cGMP regulates renal blood flow and that chronic inhibition of NO/cGMP production produces glomerular capillary hypertension, glomerular damage and proteinuria [18]. The diameter of afferent and efferent capillaries is regulated by blood flow and renal nerve activity as well as by production of NO and cGMP. Increasing cGMP levels lead to relaxation of both, afferent and efferent arterioles which has impact on glomerular filtration rates. It was shown that the cGMP-increase could counteract the angiotensin-II-induced pre-glomerular vasoconstriction [19,20] and increases renal blood flow in isolated perfused rat kidneys [21]. In addition, efferent arterioles, in which NO-synthases are expressed [3], dilate when cGMP is increased [22]. Additionally, it has been shown that inhibition of cGMP production by blocking NOS could reduce efferent blood flow [23]. Currently, it is still under investigation if cGMP increase has similar or even superior efficacy on efferent arterioles. However, overall, impaired NO/cGMP signaling reduces glomerular blood flow, whereas increasing cGMP is able to increase glomerular blood flow and especially dilatation of efferent arterioles seems to be beneficial for the prevention of glomerular damage and glomerulosclerosis [24]. Since an increase in tubular sodium excretion is sensed by the macula-densa cells and linked to vasoconstriction of afferent arterioles, reducing glomerular filtration rate, the influence of NO on this tubuloglomerular feedback (TGF) was studied as well. It was demonstrated that NO synthases are expressed in the macula densa and NOS inhibition increased TGF responses suggesting that NO could attenuate the TGF response and could therefore decrease the preglomerular vasoconstriction [25]. Nitric oxide (NO) produced by macula densa cells, modulates TGF via stimulation of sGC, leading to cGMP production and activation of PKG. Increase in NO/cGMP dilates afferent arterioles in the vicinity of macula densa, improves glomeruli function and regulates tubular osmolarity [26]. Under pathological situations such as hypertension, increase in systemic blood pressure and local glomeruli pressure provokes remodeling of kidney vascular beds. Under persistence of insults, damages become irreversible, leading to chronic kidney disease (CKD). In models of hypertension induced by several interventions (mineralocorticoid-induced hypertension by DOCA, renovascular hypertension, or angiotensin II-induced hypertension), cGMP levels were found to be significantly reduced. Increase in cGMP levels provoked vascular relaxation and reversed hypertension [27]. In the context of hypertension, local increased pressure in afferent pre-glomeruli capillaries impairs vascular membrane integrity, that induces proteinuria and provokes podocytes foot process effacement [28]. In a progressive rat model of CKD, activation of soluble guanylate cyclase (by BI 703704) increased kidney cGMP content, reduced proteinuria, decreased incidence of glomerulosclerosis and kidney interstitial lesions [29]. Besides cortical blood flow and regulation of the tone of afferent and efferent arterioles, NO/cGMP also influences medullary blood flow and vascular diameter of vasa rectae. NO-synthases are expressed in vasa recta. It was suggested that NO/cGMP driven dilatation improves medullary oxygen levels and therefore tubular function [30].

In addition to NO/cGMP mediated vasodilation from cortical glomerular capillaries to medullary blood vessels, antifibrotic, antiproliferative and anti-inflammatory effects of NO/cGMP on vascular and non-vascular compartments of the kidney are described. These additional modes of action of cGMP on renal tissues are less understood compared to the vasorelaxation, but might significantly contribute to the renoprotective effects of cGMP. Inflammation is increasingly being recognized as a driver of chronic kidney diseases, especially with diabetic disease etiology and the anti-inflammatory effects of cGMP might interrupt this early event. In the late proliferative glomerulonephritis phase, glomerular sGC protein is predominantly upregulated [31]. Increasing cGMP in mesangial cells reduces proliferation and glomerular extracellular matrix accumulation, thereby preventing glomerulonephritis [31]. Persisting renal inflammation could also lead to kidney fibrosis and remodeling resulting in chronic kidney disease or end-stage renal disease [32]. The increase in cGMP reduced renal fibrosis, decreased TGF-β, fibronectin, PAI-1, macrophage infiltration and matrix accumulation. The PDE-3 isoform is found in renal arterioles of rats and humans. In rat afferent arterioles, cAMP produced by NO interaction with the PDE-3A isoform could contribute to the vasodilatory effect of NO on renal vasculature

Damages of capillaries’ membranes and the concomitant increase in blood pressure favor infiltration of inflammatory markers such as chemokines and cytokines, which is then followed by infiltration of inflammatory cells such as macrophages or leucocytes [26]. The pro-inflammatory environment contributes to kidneys fibrosis [33] and matrix expansion (mesangial cell expansion and contraction), that ultimately decreases GFR [34]. Tubular interstitial fibrosis is found at a remarkable degree in biopsies of diabetics and CKD patients. Increasing cGMP and PKG activity increases tubular diameter, reduces macrophage tubular infiltration and reduces tubular TGF-β-induced fibrosis and apoptosis [35]. Restoring cGMP and cAMP levels could prevent end-stage renal disease in different preclinical in vitro and in vivo models associated with anti-fibrotic effects [33,36]. Although the cellular mechanisms contributing to cGMP-mediated anti-fibrotic effects are still under investigation, there is evidence from different animal models that cGMP increase prevented fibrotic remodeling of internal organs, including the kidney [32]. Thus, cGMP elevation could represent an attractive mechanism to treat kidney interstitial fibrosis in CKD patients. Among several pathways possibly involved in the anti-fibrotic effect due to cGMP elevation are stimulation cGMP-dependent protein kinases (cGK) and inhibition TGF-signaling by cGMP-dependent kinase cGKI [36].

In chronic kidney diseases mostly characterized by proteinuric and inflammatory conditions, cytoskeleton (extracellular matrix) integrity is impaired, that leads to podocyte foot process effacement [28]. Increase in cGMP levels in podocytes by selective inhibition of PDE-5 reduced proteinuria and preserved podocytes of diabetic nephropathic rats [37]. In a diabetic nephropathy animal model, reduction of serum cGMP and podocyte cGMP content was found and correlated with the loss of podocytes and glomerular membrane integrity. The treatment with selective PDE5 inhibitor (Vardenafil) increased serum cGMP levels and intracellular cGMP levels in podocytes, decreased the extent of glomerular remodeling, proteinuria, fibronectin and TGF-β expression, decreased proteinuria and attenuated podocyte damage [37]. Although the link between increase in cGMP and reduction of diabetic nephropathy is not fully understood, it is fairly suggested that restoration of cGMP levels by PDE5 inhibition in different kidney structures (podocytes and tubuli) reduces inflammation, extracellular matrix damages and thereby improve glomeruli perfusion [31,37].

In glomeruli and tubules of healthy and nephrotic rats, enzymes of cGMP production were found to be involved in the development and progression of kidney disease [38]. In preclinical rat models, increased glomerular permeability by l-NAME was reduced by NO donors or cGMP agonist (8-bromo-cGMP) [39], suggesting a direct and pivotal role of NO/sGC/cGMP in the maintenance of glomerular permeability. sGC activation by cinaciguat (BAY 58-2667) restored glomerular cGMP content, sGC expression, reduced diabetes-induced proteinuria, glomerulosclerosis and fibrosis of diabetic rats [40]. Improvement of diabetes-induced glomerular damage by sGC activation was achieved by suppression of TGF- and ERK-1 signaling. In a mouse model, sGC stimulator (BAY 41-2772), PDE5 inhibitor or PKG agonists recovered effects of angiotensin and restored podocyte permeability and loss of functionality induced by angiotensin. This effect was associated with reduced TRPC6 activity [41]. This effect is not observed in mesangial cells [42].

Regulation of salt and water hemostasis is one of the major functions of the kidney and the tubular system. There is body of evidence that tightly regulated cAMP levels influence tubular electrolyte transport but the impact of the NO/sGC system on tubular transport is only partly understood and the available data are widely descriptive.

In proximal tubules, the majority of sodium is reabsorbed, which is mainly mediated by sodium transporters, such as the sodium-hydrogen exchanger (NHE). It was shown that the activity of NHE3 on sodium retention was reduced by cGMP increase mediated by both, the NO/sGC and NP/pGC pathway [43]. More recently, it was shown that an increase of cGMP levels could counteract angiotensin-induced Na^+^-reabsorption in the proximal tubule which suggest also a blood pressure lowering mechanism independent from cGMP-mediated vascular smooth muscle relaxation [44]. The levels of tubular cyclic nucleotides like cAMP and cGMP are tightly controlled. Endogenous cGMP content is regulated on the level of cGMP production via NO-release and NO-dependent sGC stimulation. In addition, the tubular cGMP content is regulated via cGMP degrading cyclic nucleotide phosphodiesterases (PDEs). It is well established that different PDE isoenzymes are expressed along the nephron impacting on tubular cAMP and cGMP levels [45]. Moreover it was recently demonstrated that an anion transporter—called MRP4, which is a putative pump for cAMP and cGMP expressed in the proximal tubule—also influences cGMP levels [46].

In the distal tubules, it is well established that ANP tightly regulates sodium-reabsorption but the role of the NO/sGC induced cGMP production is not yet characterized. There is indirect evidence that cGMP might play a functional role here since cGMP-regulated cation-channels were identified in the DCTL of the mouse [47]. However, additional functional evidence is still missing.

In the collecting duct, cGMP derived by pGC signaling regulates the activity of the epithelial sodium channel (ENaC). However, it was also shown that NO could inhibit sodium reabsorption in isolated collecting duct preparations of rats [48]. These data were confirmed in vivo in mice lacking nNOS in the collecting duct which exhibited significantly higher sodium retention when kept on a high-salt diet compared to WT-mice [49]. These natriuretic effects of NO might be mediated also by other NO-synthases and also be sex-specific since it is only in female that collecting duct specific eNOS knockout mice, that the sodium and water secretion was impaired [50]. In the collecting duct, the effects of NO/cGMP signaling on the regulation of water channel aquaporin-2 (AQP2) need to be described here, although the effects have not been clearly understood. A previous study [51] showed that NO donors and NO synthase substrate stimulated translocation of AQP2 from cytoplasm to the plasma membrane in rat kidney slices and LLC-PK1 cells stably expressing AQP2. Unlike vasopressin, NO donor sodium nitroprusside (SNP) increased intracellular cGMP, and exogenous cGMP stimulated the membrane insertion of AQP2. In contrast, another study [52] demonstrated that ANP/cGMP decreased vasopressin-dependent AQP2 expression in the plasma membrane via a cGMP- and PKG-dependent mechanism in primary cultured inner medullary collecting duct cells of rat kidney. Activation of the NO/sGC pathway had the similar effect on AQP2 regulation as ANP. Further studies are needed to confirm the role of NO/cGMP on water reabsorption in collecting duct. Indirectly, NO/cGMP could also have impact on tubular function and sodium reabsorption by affecting renin secretion in the juxta glomerular-cells of the afferent arterioles. It has been shown that cGMP could have stimulatory and inhibitory effects on renin secretion [53]. NO could stimulate renin secretion, via cGMP-driven inhibition of PDE3 increasing cAMP levels. On the other hand, renin secretion could also be inhibited by cGMP-dependent protein kinase activity [54]. Since renin secretion is also affected by systemic vascular tone as natriuretic peptides, it might also be dependent from the specific kidney pathology.

In a rat model of hypertensive chronic kidney disease induced by NO synthase inhibition, bilateral renal denervation decreased local Renin-Angiotensin System (RAS) activity [55]. These data confirmed the direct role of sympathetic nerves in regulating kidney function in the context of hypertension. Sympathetic nerve activity (measured via neurotransmitters norepinephrine and neuropeptide Y) was found elevated during kidney diseases progression in patients and preclinical models and associated with decline in GFR [56,57,58]. Increased kidney sympathetic activity could be prevented by elevating circulating NO. Kidney hypertrophy and its progression can be experimentally stopped by renal sympathetic denervation [59].

Beside the role of cGMP/sGC/NO pathways in the development of CKD, acute kidney injury (AKI) also depends on cGMP/NO production. AKI is a pathology triggered by different types of insults that increase susceptibility to CKD development and progression [60]. Mitochondrial dysfunction [61] and inflammation [62] are increased in experimental AKI [63]. Restoration of cGMP levels by PDE inhibition [64] or by sGC activator (Cinaciguat) [65] restores mitochondrial biogenesis, restoration of cGMP accelerates recovery of kidney function after AKI [64,65]. The use of contrast agent can lead to acute kidney dysfunction [66]. Ionic contrast agents could impairs renal artery relaxation by interfering with both cAMP-mediated and cGMP-mediated pathways [67].

Increasing circulating and tissue containing cGMP/NO exerts a plethoric role in maintaining capillary relaxation (in vessels beside macula densa as well as intra glomerular), preserving integrity of kidney structures (tubules, macular densa, and podocytes) and reducing interstitial fibrosis and mesangial cells contraction and expansion as inflammation. Increasing NO/cGMP levels is foreseen as an effective treatment approach to oppose the loss of kidney structure integrity and functionality during acute and chronic kidney diseases. Therefore, hypertensive nephropathy, DKD, CKD could potentially be attenuated by such treatments, preventing kidney disease progression and end-stage renal failure.

## 6. Major Pharmacological Intervention Sites and Therapeutic Approaches on the NO/sGC Pathway

Given the broad importance of the NO/sGC/cGMP pathway for kidney health, pharmacological interventions causing cGMP increase could become an efficacious treatment option for CKD. Currently, a variety of approaches aimed to pharmacologically modify these pathways and increase cGMP are available or in under clinical and preclinical investigation. A prominent increase of cGMP could be established by either NO-supplementation by NO-donors or by direct stimulation of the sGC by using sGC stimulators and sGC activators, or by preventing cGMP degradation by blocking of PDEs. PDE5 and PDE9 are the major enzymes for cGMP-degradation and are both abundantly expressed in the kidney. Figure 1 shows the NO and NP cGMP signaling site and the major intervention sites.

### 6.1. Nitrates

Already in the 19th century, between 1860 and 1890, first descriptions for the beneficial effects of nitroglycerin (GTN) for the treatment of angina pectoris were published. Therefore, NO-donors and nitrates, which can enzymatically and non-enzymatically release NO, were the first pharmacological tools for influencing this pathway. The nitrate-derived NO binds to the heme group in the beta subunit of the sGC which triggers the formation of cGMP as described above. Nitrates are still in use for treatment of angina pectoris, however could not be used for chronic oral treatment due to physicochemical-properties, which are substantially limiting the use for chronic oral treatment regimens like they are necessary in chronic kidney diseases. In addition, nitrates could also increase reactive oxygen species which should also be avoided in disease with increased oxidative stress and endothelial dysfunction, such as diabetic kidney disease. However, these compounds are still used in preclinical settings to better understand the pathophysiology of renal diseases.

### 6.2. sGC Modulators

In contrast to NO-donors, the sGC stimulators and sGC activators are able to stimulate the sGC even in the absence of NO and have synergistic and additive effects to the endogenous NO, respectively. These compounds also bind directly to the sGC and enhancing the enzymatic activity thereby triggering the formation of cGMP out of GTP. Since cGMP plays a significant role in regulating kidney function, both sGC stimulators and sGC activators may become useful treatments for kidney diseases.

### 6.3. sGC Stimulators

sGC stimulators such as riociguat (BAY 63-2521), vericiguat (BAY 1021189) or IW-1973 bind to the sGC heterodimer sGC and stimulate the sGC even in the absence of NO. The binding site is still controversial: Initial findings indicate that the sGC stimulator BAY 41-2272 binds to the alpha-subunit of the enzyme [68], whereas data for lificiguat (YC-1) suggest a binding of sGC stimulators to the beta1-subunit of the sGC [69]. Recently, the novel sGC stimulator IWP-051 has been also shown to bind to the beta1subunit [70]. Further research is needed to clarify the exact binding mode of sGC stimulators. Nonetheless, sGC stimulators have a strong synergistic effect on cGMP production when endogenous NO is present. Scientifically, these compounds are termed as NO-independent and heme-dependent sGC stimulators. Riociguat was the first approved sGC stimulator and could be used for the treatment of pulmonary hypertension (PAH and CTEPH). The sGC stimulator vericiguat is in Phase III clinical development for the treatment of heart failure and the sGC stimulator IW-1973 entered Phase II clinical programs in hypertension, kidney diseases and heart failure.

### 6.4. sGC Activators

sGC can exist in two redox states depending on the level of oxidative stress present in the tissue. Oxidative stress leads to an oxidation of the Fe^2+^ in the heme group to Fe^3+^ and the heme group is lost subsequently which makes the enzyme insensitive to NO (Figure 2). The so-called sGC activators, such as cinaciguat (BAY 58-2667) or ataciguat (HMR 1766), are able to activate the oxidized/heme-free form; therefore, they are correctly termed as NO-independent and heme-independent activators of sGC. sGC activators bind into the heme pocket and act as NO-heme mimetics by provoking similar conformational changes as the binding of NO to heme physiological does [71]. Since most of the comorbidities in cardiovascular diseases and in chronic kidney diseases are associated with increased oxidative stress burden, leading to endothelial dysfunction with low NO production but also making the sGC insensitive to NO, it is an intriguing concept that these sGC activators could overcome this limitation by their unique mode of action. However, currently there is still a gap in our understanding at which extends low NO production and/or the occurrence of the oxidized heme free form of sGC has the most promising effect compared to other treatment approaches.

### 6.5. PDE5 and PDE9 Inhibitors

Phosphodiesterases (PDEs) cleave the major cyclic nucleotides like cAMP and cGMP in a highly efficient manner and terminate these second messengers signaling within milliseconds. PDEs, of which 11 families were identified, are different in their cellular distribution, in their regulatory domains and in their substrate specificity. Besides PDE6, which is localized in the eye, PDE5 and PDE9 are the main cGMP degrading enzymes. PDE5 and PDE9 are the major enzymes for cGMP-degradation and are both abundantly expressed in the kidney and blocking of PDE5 and PDE9 may effectively target also renal tissues.

PDE5 Inhibitors (PDE5i) were introduced into medical therapy for the treatment of erectile dysfunction (ED) in 1998 when sildenafil was launched (Viagra™), followed by vardenafil (Levitra™) and tadalafil (Cialis™) in 2003. In 2007 and 2009, sildenafil and tadalafil were also approved for the treatment of pulmonary arterial hypertension (PAH) as Revatio™ and Adcirca™, respectively. PDE9 Inhibitors are not launched for any indication. However, it was shown preclinical that PDE9 inhibitors increased learning and memory in animal models and had some treatment effects in dementia models. Therefore, currently clinical testing is ongoing in Alzheimer dementia.

## 7. Reinforcing the NO/sGC/cGMP Axis as Treatment Option for Kidney Diseases

In recent years, many preclinical studies assessed the physiological effects of cGMP-increasing mechanisms not only on physiological kidney function but investigated the pharmacological and therapeutic impact on kidney diseases. NO-donors, PDE5 inhibitors and sGC stimulators and sGC activators were studied in preclinical models and an overview on these studies is summarized in Table 1. Homoarginine-induced vascular calcification, in addition to hyperphosphatemia present during chronic kidney disease, is a well-known risk factor for cardiovascular disease. Molsidomine is an NO donor that works by releasing NO by its active metabolite linsidomine. It is currently used as an orally active, long-lasting vasodilator that is clinically approved for the treatment of angina. One study showed that treatment with molsidomine significantly reduced calcification of the aortas and markers of osteogenesis [72]. In another rat model of cardiorenal failure, treatment with molsidomine improved creatinine clearance and cardiac function and also displayed a mild reduction in blood pressure [73]. Molsidomine treatment was also shown to reduce renal pathological burden by reducing proteinuria, podocyte stress and renal injury in hypercholesteremic rats [74]. Furthermore, in a recent study using two animal models of CKD, it was shown that treatment with another long-lasting NO donor, *S*-nitrosated human serum albumin (SNO-HSA), displayed a dual beneficial effect, as it was able to increase hematopoiesis as well as effectively reduce renal fibrosis [75]. Although NO-donors might exert some beneficial effects on kidney function, their use is also limited by the prominent reduction of blood pressure and more studies are needed to carefully assess the window between desired kidney protective effect and undesired hypotension.

Along these lines, phosphodiesterase inhibitors do show only moderate effects on systemic blood pressure and are well tolerated drugs. While there is almost no preclinical evidence evaluating the therapeutic effects of PDE9 inhibitors in kidney disease, several studies have demonstrated that treatment with PDE5 inhibitors is reno-protective. In a rabbit model of acute kidney injury, sildenafil was shown to reduce renal histopathology and electrolyte derangement while showing an improvement in creatinine clearance. Furthermore, it is well known that a majority of CKD patients also suffer from hypertension, and therefore targeting the antihypertensive effects of PDE5 inhibitors would be additionally beneficial in reducing kidney disease burden. Several studies using rodent models of renovascular hypertension have shown that PDE5 inhibitors such as sildenafil or vardenafil were able to cause significant decreases in blood pressure compared to administration of saline [86,89]. In addition, following treatment with the PDE5 inhibitor the animals also showed increased baroreceptor sensitivity and decreased oxidative stress. Interestingly, apart from cardio-renal pathologies, a PDE5 siRNA was also shown to inhibit proliferation and survival of human renal carcinoma cells [88].

As mentioned above, the effects of inhibition of cGMP degradation is limited in diseases with impaired endothelial function and low endogenous cGMP production. Therefore, NO-independent sGC stimulators and sGC activators might be even more beneficial. The effect of sGC stimulators and sGC activators on the kidneys was comprehensively summarized in a review by Stasch, J.P. et al., 2015 [76]. In numerous studies, in disease models of different etiologies, ranging from hypertensive-induced renal damage to CKD driven by inflammation, diabetes or obesity, sGC stimulators and sGC activators have consistently shown renoprotective effects. More recently, sGC stimulators and sGC activators were also investigated in the so called ZSF-1 rat, a model which is characterized by hypertension, obesity and type-2-diabetes. The sGC stimulator IW-1973 and the sGC activators have shown efficacy there and were most effective in blood pressure-lowering dosages [29]. Currently, it is not yet clear if sGC stimulators and sGC activators have different effects and efficacy in kidney diseases independent from the etiology and comorbidities. However, in essence, these molecules seem to have a huge potential for the treatment of kidney diseases. 

## 8. Outlook

Together, NO/sGC/cGMP plays a pivotal role for the regulation of kidney function by regulation of the kidney blood flow as well as by protective effects on glomerular and tubular compartments. Numerous preclinical studies have shown that targeting the NO/sGC/cGMP signaling cascade and increasing cGMP production are beneficial in various kidney pathologies. In the future, it will become very interesting to see if the compelling preclinical findings with sGC stimulators and sGC activators in kidney disease animal models can be successfully translated into clinical studies in kidney disease patients. First studies, using Nitrates and PDE inhibitors in rather small populations, were not fully conclusive. However, currently, a Phase 2 clinical study with an sGC stimulators in ongoing in CKD patients (NCT03217591) and sGC activators have also shown a promising preclinical profile [28] and might also be tested clinically in the future. Given the unique mode of action of sGC stimulators and sGC activators, these treatment approaches might hopefully provide an efficacious treatment alternative for kidney diseases in the future. Since the NO/sGC/cGMP system also actively regulates endothelial as well as myocardial functions, these drugs might have an even broader treatment potential in cardio-renal indications, by targeting the kidney, the heart and the vasculature.

## Figures and Tables

**Table 1:** Current preclinical evidence for beneficial effects of cGMP-based treatments with relevance in kidney disease.

Citation	Compound	Disease	Treatment Groups	Results
***NO donors***				
Oshiro, S. et al., 2018 [75]	*S*-nitrosated human serum albumin (SNO-HSA)	Chronic kidney disease	*Cisplatin-induced renal anemia model* Saline SNO-HSA (240 nmol/kg of NO) *Urinary ureter obstruction model* Saline SNO-HSA (48 nmol/mouse of NO)	Treatment of animals with SNO-HSA
Alesutan, I. et al., 2016 [72]	Molsidomine	Vascular calcification in chronic kidney disease	Homoarginine + control Homoarginine + molsidomine (120 mg/L)	Molsidomine treatment reversed homoarginine-induced vascular calcification in mice compared to control treatment
Bongartz, L.G. et al., 2010 [73]	Molsidomine	Chronic kidney disease	Vehicle Molsidomine (120 mg/L)	Treatment with molsidomine resulted in a mild reduction in blood pressure and improved creatinine clearance. Furthermore, molsidomine treatment improved cardiac function.
Attia, D.M. et al., 2002 [74]	Molsidomine	Hypercholesterolemia	Vehicle Molsidomine (120 mg/L)	In hypercholesteremic rats, treatment with molsidomine reduces proteinuria and podocyte stress as well as glomerular and tubulo-interstitial injury compared to rats treated with the vehicle
***sGC stimulators & sGC activators***				
Stasch, J.P. et al., 2015 [76]	sGC stimulators and sGC activators	Review summarizing the effects of sGC stimulators and activators in preclinical models of kidney disease in papers published between 2004 and 2012.		
Tobin et al., 2018 [77]	IW-1973	Hypertension-induced kidney disease	Dahl rats + Vehicle Dahl rats + IW-1973 (1–10 mg/kg/d)	Treatment with IW-1973 reduced blood pressure prevented the progression of proteinuria and reduced renal fibrosis and markers of renal inflammation
Follmann, M. et al., 2017 [78]	Vericiguat	Chronic heart failure	Renin TG rats + L-NAME + Placebo Renin TG rats + L-NAME + Vericiguat (3 mg/kg) Renin TG rats + L-NAME + Vericiguat (10 mg/kg)	Treatment with vericiguat caused a reduction in KIM-1 and osteopontin expression, we all as a dose-dependent reduction in proteinuria
Profy, A.V. et al., 2018 [79]	IW-1973	Diabetic nephropathy	Obese ZSF-1 rats + Vehicle Obese ZSF-1 rats + IW-1973 (1–10 mg/kg/d)	Treatment with IW-1973 reduced kidney weight, proteinuria, urine volume and fasting glucose levels.
Schinner, E. et al., 2017 [36]	BAY 41-8543	Renal fibrosis post Unilateral Ureter Obstruction	Unilateral Ureter Obstruction (UUO) WT UUO WT + BAY 41-8543 UUO cGKI-KO UUO cGKI-KO + BAY 41-8543	Post unilateral ureter obstruction, BAY41-8543 reduces mRNA expression of several biomarkers of fibrosis in the kidney of WT mice whereas the expression in the cGKI-KO remains unchanged
Cunha, V.D. et al., 2016 [80]	MRL-001	Chronic kidney disease	ZSF-1 rats + Vehicle ZSF-1 rats + MRL-001 (1 mg/kg/d)	Compared to vehicle, treatment with MRL-001 attenuated markers of diabetic nephropathy, tubular damage, proteinuria and oxidative stress, and improved glucose tolerance
Boustany-Kari, C.M. et al., 2015 [29]	BI 703704	Diabetic nephropathy	Obese ZSF-1 rats + Vehicle Obese ZSF-1 rats + BI 703704 (0.3–10 mg/kg/d)	Treatment with the sGC activator, BI 703704, reduced protein excretion, glomerulosclerosis and renal interstitial lesions in a dose-dependent manner, however it had an effect on BP and HR only with 10 mg/kg/d of BI 703704
Stancu, B. et al., 2015 [81]	BAY 41-8543	Arterial wall remodeling in a model of mild uremia	Sham Subtotally nephrectomized (SNX) SNX + BAY 41-8543 SNX + hydralazine	BAY 41-8543 ameliorates uremic aortic remodeling and stiffness in a blood-pressure independent manner
Nagasu, H. et al., 2012 [82]	BAY 41-2272	Kidney injury post unilateral nephrectomy	Sham WT Uninephrectomy WT Sham eNOS KO Uninephrectomy eNOS KO Sham eNOS KO + BAY 41-2272 Uninephrectomy eNOS KO + BAY 41-2272 hPTECs treated with BAY 41-2272 hPTECs treated with GSNO	BAY 41-2272 induces compensatory renal hypertrophy and protects renal function in uninephrectomized eNOS KO mice Both BAY 41-2272 and GSNO activate the Akt-mToR pathway in hPTECs and are thus able to stimulate protein synthesis in proximal tubules
Sharma, M. et al., 2009 [83]	BAY 41-2272	Chronic kidney disease	Vehicle ADMA ADMA + BAY 41-2272	Bay 41-2272 attenuated ADMA-induced increases in albumin permeability in isolated glomeruli
Boerrigter, G. et al., 2003 [84]	BAY 41-2272 and nitroglycerin	Congestive heart failure	BAY 41-2272 (2 µg/kg/min) BAY 41-2272 (10 µg/kg/min) Nitroglycerin (1 µg/kg/min) Nitroglycerin (5 µg/kg/min)	Both, the administration of high dose BAY 41-2272 and nitroglycerin reduced mean arterial pressure, increased cardiac output and renal blood flow and maintain glomerular filtration rate. In addition, nitroglycerin treatment decreased right arterial pressure and pulmonary vascular resistance.
***PDE inhibitors***				
Ramseyer, V.D. et al., 2016 [85]	Vardenafil	Angiotensin II-induced hypertension	Saline-treated rats + vardenafil Ang II-treated rats + vardenafil	Treatment with vardenafil restores NO-mediated inhibition of NKCC2 activity and stimulation of cGMP production in isolated thick ascending limbs from Ang II-treated rats
Cavalcanti, C.O. et al., 2016 [86]	Sildenafil	Renovascular hypertension	2-kidney-1-clip (2K1C) 2K1C + Sildenafil	Hypertensive rats treated with sildenafil had increased baroreflex sensitivity, decreased oxidative stress, were protected from autonomic imbalance and had an overall reduction in BP independent of changes in HR
Lauver, D.A. et al., 2014 [87]	Sildenafil	Contrast induced acute kidney injury (CIAKI)	CIAKI CIAKI + sildenafil (6 mg/kg)	Treatment with sildenafil in a rabbit model of CIAKI resulted in decreased levels of kidney histopathology, serum creatinine and electrolyte derangement.
Ren, Y. et al., 2014 [88]	PDE5 siRNA	Renal carcinoma	PDE5 siRNA	Suppression of PDE5 expression with PDE5 siRNA inhibits proliferation and survival of human renal carcinoma cells in vitro
Stegbauer, J. et al., 2013 [89]	Sildenafil	Renovascular hypertension	Wild type + 2KIC + sildenafil (100 mg/kg/d) NO GC1 KO + 2K1C + sildenafil	Sildenafil significantly reduced blood pressure in WT mice induced with 2K1C-hypertension, compared to NO GC1 KO mice.
Whitaker, R.M. et al., 2013 [64]	PDE3, PDE4 and PDE5 inhibitors	Acute kidney injury	In vitro Renal tubular epithelial cells treated with PDE3, PDE4 and PDE5 inhibitors In vivo Folic acid (FA)-induced AKI FA-induced AKI + sildenafil (0.3 mg/kg)	In vitro treatment with PDE3 and PDE5 inhibitors, but not PDE4 inhibitors induce mitochondrial biogenesis in renal proximal tubular epithelial cells, suggesting restoration of mitochondrial function post AKI. In addition, sildenafil treatment in mice with AKI also showed signs of mitochondrial biogenesis in the renal cortex

## References

[1] Beuve A. (2016). Thiol-based redox modulation of soluble guanylyl cyclase, the nitric oxide receptor. Antioxid. Redox Signal..

[2] Rahaman M.M., Nguyen A.T., Miller M.P., Hahn S.A., Sparacino-Watkins C., Jobbagy S., Carew N.T., Cantu-Medellin N., Wood K.C., Baty C.J. (2017). Cytochrome b5 reductase 3 modulates soluble guanylate cyclase redox state and cgmp signaling. Circ. Res..

[3] Bachmann S., Bosse H.M., Mundel P. (1995). Topography of nitric oxide synthesis by localizing constitutive NO synthases in mammalian kidney. Am. J. Physiol..

[4] Lee D.L., Sasser J.M., Hobbs J.L., Boriskie A., Pollock D.M., Carmines P.K., Pollock J.S. (2005). Posttranslational regulation of NO synthase activity in the renal medulla of diabetic rats. Am. J. Physiol. Renal Physiol..

[5] Sasser J.M., Brinson K.N., Tipton A.J., Crislip G.R., Sullivan J.C. (2015). Blood pressure, sex, and female sex hormones influence renal inner medullary nitric oxide synthase activity and expression in spontaneously hypertensive rats. J. Am. Heart Assoc..

[6] Mundel P., Gambaryan S., Bachmann S., Koesling D., Kriz W. (1995). Immunolocalization of soluble guanylyl cyclase subunits in rat kidney. Histochem. Cell Biol..

[7] Theilig F., Bostanjoglo M., Pavenstadt H., Grupp C., Holland G., Slosarek I., Gressner A.M., Russwurm M., Koesling D., Bachmann S. (2001). Cellular distribution and function of soluble guanylyl cyclase in rat kidney and liver. J. Am. Soc. Nephrol..

[8] Wolfertstetter S., Huettner J.P., Schlossmann J. (2013). cGMP-dependent protein kinase inhibitors in health and disease. Pharmaceuticals.

[9] Buglioni A., Burnett J.C. (2015). Pathophysiology and the cardiorenal connection in heart failure. Circulating hormones: Biomarkers or mediators. Clin. Chim. Acta.

[10] Silver M.A. (2006). The natriuretic peptide system: Kidney and cardiovascular effects. Curr. Opin. Nephrol. Hypertens..

[11] Brenner B.M. (2004). The Kidney.

[12] Ohishi K., Carmines P.K., Inscho E.W., Navar L.G. (1992). EDRF-angiotensin II interactions in rat juxtamedullary afferent and efferent arterioles. Am. J. Physiol. Renal Physiol..

[13] Simons M. (2018). The benefits of tubular proteinuria: An evolutionary perspective. J. Am. Soc. Nephrol..

[14] Zoja C., Abbate M., Remuzzi G. (2015). Progression of renal injury toward interstitial inflammation and glomerular sclerosis is dependent on abnormal protein filtration. Nephrol. Dial. Transplant..

[15] Liu G.-L., Liu L., Barajas L. (1996). Development of NOS-containing neuronal somata in the rat kidney. J. Auton. Nerv. Syst..

[16] Friebe A., Sandner P., Schmidtko A. (2017). Meeting report of the 8th International Conference on cGMP “cGMP: Generators, effectors, and therapeutic implications” at Bamberg, Germany, from June 23 to 25, 2017. Naunyn Schmiedebergs Arch. Pharmacol..

[17] Sandner P. (2018). From molecules to patients: Exploring the therapeutic role of soluble guanylate cyclase stimulators. Biol. Chem..

[18] Baylis C., Qiu C. (1996). Importance of nitric oxide in the control of renal hemodynamics. Kidney Int..

[19] Patzak A., Kleinmann F., Lai E.Y., Kupsch E., Skelweit A., Mrowka R. (2004). Nitric oxide counteracts angiotensin II induced contraction in efferent arterioles in mice. Acta Physiol. Scand..

[20] Patzak A., Persson A.E. (2007). Angiotensin II-nitric oxide interaction in the kidney. Curr. Opin. Nephrol. Hypertens..

[21] Sandner P., Kornfeld M., Ruan X., Arendshorst W.J., Kurtz A. (1999). Nitric oxide/cAMP interactions in the control of rat renal vascular resistance. Circ. Res..

[22] Schricker K., Ritthaler T., Krämer B.K., Kurtz A. (1993). Effect of endothelium-derived relaxing factor on renin secretion from isolated mouse renal juxtaglomerular cells. Acta Physiol. Scand..

[23] Polichnowski A.J., Licea-Vargas H., Picken M., Long J., Bisla R., Williamson G.A., Bidani A.K., Griffin K.A. (2015). Glomerulosclerosis in the diet-induced obesity model correlates with sensitivity to nitric oxide inhibition but not glomerular hyperfiltration or hypertrophy. Am. J. Physiol. Renal Physiol..

[24] Bidani A.K., Polichnowski A.J., Loutzenhiser R., Griffin K.A. (2013). Renal microvascular dysfunction, hypertension and CKD progression. Curr. Opin. Nephrol. Hypertens..

[25] Thorup C., Erik A., Persson G. (1996). Macula densa derived nitric oxide in regulation of glomerular capillary pressure. Kidney Int..

[26] Evans L.C., Petrova G., Kurth T., Yang C., Bukowy J.D., Mattson D.L., Cowley A.W. (2017). Increased perfusion pressure drives renal t-cell infiltration in the dahl salt-sensitive rat. Hypertension.

[27] Otsuka Y., DiPiero A., Hirt E., Brennaman B., Lockette W. (1988). Vascular relaxation and cGMP in hypertension. Am. J. Physiol. Heart Circ. Physiol..

[28] Suleiman H.Y., Roth R., Jain S., Heuser J.E., Shaw A.S., Miner J.H. (2017). Injury-induced actin cytoskeleton reorganization in podocytes revealed by super-resolution microscopy. JCI Insight.

[29] Boustany-Kari C.M., Harrison P.C., Chen H., Lincoln K.A., Qian H.S., Clifford H., Wang H., Zhang X., Gueneva-Boucheva K., Bosanac T. (2016). A soluble guanylate cyclase activator inhibits the progression of diabetic nephropathy in the ZSF1 rat. J. Pharmacol. Exp. Ther..

[30] Brezis M., Heyman S.N., Dinour D., Epstein F.H., Rosen S. (1991). Role of nitric oxide in renal medullary oxygenation. Studies in isolated and intact rat kidneys. J. Clin. Investig..

[31] Hohenstein B., Daniel C., Wagner A., Stasch J.P., Hugo C. (2005). Stimulation of soluble guanylyl cyclase inhibits mesangial cell proliferation and matrix accumulation in experimental glomerulonephritis. Am. J. Physiol. Renal Physiol..

[32] Schinner E., Wetzl V., Schlossmann J. (2015). Cyclic nucleotide signalling in kidney fibrosis. Int. J. Mol. Sci..

[33] Sandner P., Berger P., Zenzmaier C. (2017). The potential of sGC modulators for the treatment of age-related fibrosis: A mini-review. Gerontology.

[34] Abboud H.E. (2012). Mesangial cell biology. Exp. Cell Res..

[35] Cui W., Maimaitiyiming H., Qi X., Norman H., Zhou Q., Wang X., Fu J., Wang S. (2014). Increasing cGMP-dependent protein kinase activity attenuates unilateral ureteral obstruction-induced renal fibrosis. Am. J. Physiol. Renal Physiol..

[36] Schinner E., Wetzl V., Schramm A., Kees F., Sandner P., Stasch J.P., Hofmann F., Schlossmann J. (2017). Inhibition of the TGFβ signalling pathway by cGMP and cGMP-dependent kinase I in renal fibrosis. FEBS Open Bio.

[37] Fang L., Radovits T., Szabo G., Mozes M.M., Rosivall L., Kokeny G. (2013). Selective phosphodiesterase-5 (PDE-5) inhibitor vardenafil ameliorates renal damage in type 1 diabetic rats by restoring cyclic 3′, 5′ guanosine monophosphate (cGMP) level in podocytes. Nephrol. Dial Transplant..

[38] Helwig J.J., Yusufi A.N., Rebel G., Geiser J., Bollack C., Mandel P. (1980). Distribution of enzymes of cGMP metabolism in glomeruli and tubules isolated from normal and nephrotic rat kidney cortex. Int. J. Biochem..

[39] Dolinina J., Sverrisson K., Rippe A., Oberg C.M., Rippe B. (2016). Nitric oxide synthase inhibition causes acute increases in glomerular permeability in vivo, dependent upon reactive oxygen species. Am. J. Physiol. Renal Physiol..

[40] Czirok S., Fang L., Radovits T., Szabó G., Szénási G., Rosivall L., Merkely B., Kökény G. (2017). Cinaciguat ameliorates glomerular damage by reducing ERK1/2 activity and TGF-ß expression in type-1 diabetic rats. Sci. Rep..

[41] Hall G., Rowell J., Farinelli F., Gbadegesin R.A., Lavin P., Wu G., Homstad A., Malone A., Lindsey T., Jiang R. (2014). Phosphodiesterase 5 inhibition ameliorates angiontensin II-induced podocyte dysmotility via the protein kinase G-mediated downregulation of TRPC6 activity. Am. J. Physiol. Renal Physiol..

[42] Tack I., Marin Castano E., Pecher C., Praddaude F., Bascands J.L., Bompart G., Ader J.L., Girolami J.P. (1997). Endothelin increases NO-dependent cGMP production in isolated glomeruli but not in mesangial cells. Am. J. Physiol..

[43] Eitle E., Hiranyachattada S., Wang H., Harris P.J. (1998). Inhibition of proximal tubular fluid absorption by nitric oxide and atrial natriuretic peptide in rat kidney. Am. J. Physiol..

[44] Shirai A., Yamazaki O., Horita S., Nakamura M., Satoh N., Yamada H., Suzuki M., Kudo A., Kawakami H., Hofmann F. (2014). Angiotensin II dose-dependently stimulates human renal proximal tubule transport by the nitric oxide/guanosine 3′, 5′-cyclic monophosphate pathway. J. Am. Soc. Nephrol..

[45] Dousa T.P. (1999). Cyclic-3′, 5′-nucleotide phosphodiesterase isozymes in cell biology and pathophysiology of the kidney. Kidney Int..

[46] Van Aubel R.A., Smeets P.H., Peters J.G., Bindels R.J., Russel F.G. (2002). The MRP4/ABCC4 gene encodes a novel apical organic anion transporter in human kidney proximal tubules: Putative efflux pump for urinary cAMP and cGMP. J. Am. Soc. Nephrol..

[47] Ciampolillo F., McCoy D.E., Green R.B., Karlson K.H., Dagenais A., Molday R.S., Stanton B.A. (1996). Cell-specific expression of amiloride-sensitive, Na(+)-conducting ion channels in the kidney. Am. J. Physiol. Cell Physiol..

[48] Stoos B.A., Garcia N.H., Garvin J.L. (1995). Nitric oxide inhibits sodium reabsorption in the isolated perfused cortical collecting duct. J. Am. Soc. Nephrol..

[49] Hyndman K.A., Mironova E.V., Giani J.F., Dugas C., Collins J., McDonough A.A., Stockand J.D., Pollock J.S. (2017). Collecting duct nitric oxide synthase 1ß activation maintains sodium homeostasis during high sodium intake through suppression of aldosterone and renal angiotensin II pathways. J. Am. Heart Assoc..

[50] Gao Y., Stuart D., Pollock J.S., Takahishi T., Kohan D.E. (2016). Collecting duct-specific knockout of nitric oxide synthase 3 impairs water excretion in a sex-dependent manner. Am. J. Physiol. Renal Physiol..

[51] Bouley R., Breton S., Sun T.-X., McLaughlin M., Nsumu N.N., Lin H.Y., Ausiello D.A., Brown D. (2000). Nitric oxide and atrial natriuretic factor stimulate cGMP-dependent membrane insertion of aquaporin 2 in renal epithelial cells. J. Clin. Investig..

[52] Klokkers J., Langehanenberg P., Kemper B., Kosmeier S., Bally G.V., Riethmüller C., Wunder F., Sindic A., Pavenstädt H., Schlatter E. (2009). Atrial natriuretic peptide and nitric oxide signaling antagonizes vasopressin-mediated water permeability in inner medullary collecting duct cells. Am. J. Physiol. Renal Physiol..

[53] Kurtz A. (2011). Renin release: Sites, mechanisms, and control. Annu. Rev. Physiol..

[54] Wagner C., Pfeifer A., Ruth P., Hofmann F., Kurtz A. (1998). Role of cGMP-kinase II in the control of renin secretion and renin expression. J. Clin. Investig..

[55] Eriguchi M., Tsuruya K., Haruyama N., Yamada S., Tanaka S., Suehiro T., Noguchi H., Masutani K., Torisu K., Kitazono T. (2015). Renal denervation has blood pressure-independent protective effects on kidney and heart in a rat model of chronic kidney disease. Kidney Int..

[56] Armenia A., Munavvar A.S., Abdullah N.A., Helmi A., Johns E.J. (2004). The contribution of adrenoceptor subtype(s) in the renal vasculature of diabetic spontaneously hypertensive rats. Br. J. Pharmacol..

[57] Nakamori H., Yoshida S.I., Ishiguro H., Suzuki S., Yasuzaki H., Hashimoto T., Ishigami T., Hirawa N., Toya Y., Umemura S. (2017). Arterial wall hypertrophy is ameliorated by alpha2-adrenergic receptor antagonist or aliskiren in kidneys of angiotensinogen-knockout mice. Clin. Exp. Nephrol..

[58] Zoccali C., D’Arrigo G., Leonardis D., Pizzini P., Postorino M., Tripepi G., Mallamaci F., van den Brand J., van Zuilen A., Wetzels J. (2018). Neuropeptide Y and chronic kidney disease progression: A cohort study. Nephrol. Dial Transplant..

[59] Polhemus D.J., Trivedi R.K., Gao J., Li Z., Scarborough A.L., Goodchild T.T., Varner K.J., Xia H., Smart F.W., Kapusta D.R. (2017). Renal sympathetic denervation protects the failing heart via inhibition of neprilysin activity in the kidney. J. Am. Coll. Cardiol..

[60] Basile D.P., Bonventre J.V., Mehta R., Nangaku M., Unwin R., Rosner M.H., Kellum J.A., Ronco C. (2016). Progression after AKI: Understanding maladaptive repair processes to predict and identify therapeutic treatments. J. Am. Soc. Nephrol..

[61] Whitaker R.M., Stallons L.J., Kneff J.E., Alge J.L., Harmon J.L., Rahn J.J., Arthur J.M., Beeson C.C., Chan S.L., Schnellmann R.G. (2015). Urinary mitochondrial DNA is a biomarker of mitochondrial disruption and renal dysfunction in acute kidney injury. Kidney Int..

[62] Rabb H., Griffin M.D., McKay D.B., Swaminathan S., Pickkers P., Rosner M.H., Kellum J.A., Ronco C. (2016). Inflammation in AKI: Current understanding, key questions, and knowledge gaps. J. Am. Soc. Nephrol..

[63] Matejovic M., Ince C., Chawla L.S., Blantz R., Molitoris B.A., Rosner M.H., Okusa M.D., Kellum J.A., Ronco C. (2016). Renal hemodynamics in AKI: In search of new treatment targets. J. Am. Soc. Nephrol..

[64] Whitaker R.M., Wills L.P., Stallons L.J., Schnellmann R.G. (2013). cGMP-selective phosphodiesterase inhibitors stimulate mitochondrial biogenesis and promote recovery from acute kidney injury. J. Pharmacol. Exp. Ther..

[65] Whitaker R.M., Corum D., Beeson C.C., Schnellmann R.G. (2016). Mitochondrial biogenesis as a pharmacological target: A new approach to acute and chronic diseases. Annu. Rev. Pharmacol. Toxicol..

[66] Golshahi J., Nasri H., Gharipour M. (2014). Contrast-induced nephropathy: A literature review. J. Nephropathol..

[67] Discigil B., Evora P.R.B., Pearson P.J., Viaro F., Rodrigues A.J., Schaff H.V. (2004). Ionic radiocontrast inhibits endothelium-dependent vasodilation of the canine renal artery in vitro: Possible mechanism of renal failure following contrast medium infusion. Braz. J. Med. Biol. Res..

[68] Stasch J.P., Becker E.M., Alonso-Alija C., Apeler H., Dembowsky K., Feurer A., Gerzer R., Minuth T., Perzborn E., Pleiss U. (2001). NO-independent regulatory site on soluble guanylate cyclase. Nature.

[69] Denninger J.W., Schelvis J.P., Brandish P.E., Zhao Y., Babcock G.T., Marletta M.A. (2000). Interaction of soluble guanylate cyclase with YC-1: Kinetic and resonance Raman studies. Biochemistry.

[70] Wales J.A., Chen C.Y., Breci L., Weichsel A., Bernier S.G., Sheppeck J.E., Solinga R., Nakai T., Renhowe P.A., Jung J. (2018). Discovery of stimulator binding to a conserved pocket in the heme domain of soluble guanylyl cyclase. J. Biol. Chem..

[71] Martin F., Baskaran P., Ma X., Dunten P.W., Schaefer M., Stasch J.P., Beuve A., van den Akker F. (2010). Structure of cinaciguat (BAY 58–2667) bound to Nostoc H-NOX domain reveals insights into heme-mimetic activation of the soluble guanylyl cyclase. J. Biol. Chem..

[72] Alesutan I., Feger M., Tuffaha R., Castor T., Musculus K., Buehling S.S., Heine C.L., Kuro O.M., Pieske B., Schmidt K. (2016). Augmentation of phosphate-induced osteo-/chondrogenic transformation of vascular smooth muscle cells by homoarginine. Cardiovasc. Res..

[73] Bongartz L.G., Braam B., Verhaar M.C., Cramer M.J., Goldschmeding R., Gaillard C.A., Steendijk P., Doevendans P.A., Joles J.A. (2010). The nitric oxide donor molsidomine rescues cardiac function in rats with chronic kidney disease and cardiac dysfunction. Am. J. Physiol. Heart Circ. Physiol..

[74] Attia D.M., Ni Z.N., Boer P., Attia M.A., Goldschmeding R., Koomans H.A., Vaziri N.D., Joles J.A. (2002). Proteinuria is preceded by decreased nitric oxide synthesis and prevented by a NO donor in cholesterol-fed rats. Kidney Int..

[75] Oshiro S., Ishima Y., Maeda H., Honda N., Bi J., Kinoshita R., Ikeda M., Iwao Y., Imafuku T., Nishida K. (2018). Dual therapeutic effects of an albumin-based nitric oxide donor on 2 experimental models of chronic kidney disease. J. Pharm. Sci..

[76] Stasch J.P., Schlossmann J., Hocher B. (2015). Renal effects of soluble guanylate cyclase stimulators and activators: A review of the preclinical evidence. Curr. Opin. Pharmacol..

[77] Tobin J.V., Zimmer D.P., Shea C., Germano P., Bernier S.G., Liu G., Long K., Miyashiro J., Ranganath S., Jacobson S. (2018). Pharmacological characterization of iw-1973, a novel soluble guanylate cyclase stimulator with extensive tissue distribution, anti-hypertensive, anti-inflammatory, and anti-fibrotic effects in preclinical models of disease. J. Pharmacol. Exp. Ther..

[78] Follmann M., Ackerstaff J., Redlich G., Wunder F., Lang D., Kern A., Fey P., Griebenow N., Kroh W., Becker-Pelster E.M. (2017). Discovery of the soluble guanylate cyclase stimulator vericiguat (BAY 1021189) for the treatment of chronic heart failure. J. Med. Chem..

[79] Profy A.T., Shea C., Lonie E., Liu G., Milne G.T., Currie M.G., Masferrer J.L. (2017). IW-1973, a Soluble Guanylate Cyclase Stimulator, Inhibits Progression of Diabetic Nephropathy in the ZSF1 Rat Model.

[80] Valdeci da Cunha K.S., Price O., Sinz C.J., Kim R.M. (2016). Activation of soluble guanylate cyclase ameliorates the progression of glucose intolerance and nephropathy in obese ZSF1 rats. Circulation.

[81] Stancu B., Kramer S., Loof T., Mika A., Amann K., Neumayer H.H., Peters H. (2015). Soluble guanylate cyclase stimulator BAY 41–8543 and female sex ameliorate uremic aortic remodeling in a rat model of mild uremia. J. Hypertens..

[82] Nagasu H., Satoh M., Kidokoro K., Nishi Y., Channon K.M., Sasaki T., Kashihara N. (2012). Endothelial dysfunction promotes the transition from compensatory renal hypertrophy to kidney injury after unilateral nephrectomy in mice. Am. J. Physiol. Renal Physiol..

[83] Sharma M., Zhou Z., Miura H., Papapetropoulos A., McCarthy E.T., Sharma R., Savin V.J., Lianos E.A. (2009). ADMA injures the glomerular filtration barrier: Role of nitric oxide and superoxide. Am. J. Physiol. Renal Physiol..

[84] Boerrigter G., Costello-Boerrigter L.C., Cataliotti A., Tsuruda T., Harty G.J., Lapp H., Stasch J.P., Burnett J.C. (2003). Cardiorenal and humoral properties of a novel direct soluble guanylate cyclase stimulator BAY 41–2272 in experimental congestive heart failure. Circulation.

[85] Ramseyer V.D., Ortiz P.A., Carretero O.A., Garvin J.L. (2016). Angiotensin II-mediated hypertension impairs nitric oxide-induced NKCC2 inhibition in thick ascending limbs. Am. J. Physiol. Renal Physiol..

[86] Cavalcanti C.O., Alves R.R., de Oliveira A.L., Cruz J.C., de Franca-Silva M.S., Braga V.A., Balarini C.M. (2016). Inhibition of PDE5 restores depressed baroreflex sensitivity in renovascular hypertensive rats. Front. Physiol..

[87] Lauver D.A., Carey E.G., Bergin I.L., Lucchesi B.R., Gurm H.S. (2014). Sildenafil citrate for prophylaxis of nephropathy in an animal model of contrast-induced acute kidney injury. PLoS ONE.

[88] Ren Y., Zheng J., Yao X., Weng G., Wu L. (2014). Essential role of the cGMP/PKG signaling pathway in regulating the proliferation and survival of human renal carcinoma cells. Int. J. Mol. Med..

[89] Stegbauer J., Friedrich S., Potthoff S.A., Broekmans K., Cortese-Krott M.M., Quack I., Rump L.C., Koesling D., Mergia E. (2013). Phosphodiesterase 5 attenuates the vasodilatory response in renovascular hypertension. PLoS ONE.

